# Molecular surveillance of artemisinin resistance-related *Pfk13* and *pfcrt* polymorphisms in imported *Plasmodium falciparum* isolates reported in eastern China from 2015 to 2019

**DOI:** 10.1186/s12936-022-04398-x

**Published:** 2022-12-04

**Authors:** Xiangli Kong, Jun Feng, Yan Xu, Ge Yan, Shuisen Zhou

**Affiliations:** 1 National Institute of Parasitic Diseases, Chinese Center for Disease Control and Prevention (Chinese Center for Tropical Diseases Research); NHC Key Laboratory of Parasite and Vector Biology; WHO Collaborating Centre for Tropical Diseases; National Center for International Research on Tropical Diseases, Shanghai, People’s Republic of China; 2 Shandong Institute of Parasitic Diseases, Shandong First Medical University & Shandong Academy of Medical Sciences, Jining, People’s Republic of China; 3 Shanghai Municipal Center for Disease Control and Prevention, Shanghai, People’s Republic of China

**Keywords:** *Plasmodium falciparum*, *pfk13*, *Pfcrt*, Antimalarial drug resistance, Africa, Shandong

## Abstract

**Background:**

Artemisinin-based combination therapy (ACT) has been recommended as the first-line treatment by the World Health Organization to treat uncomplicated *Plasmodium falciparum* malaria. However, the emergence and spread of *P. falciparum* resistant to artemisinins and their partner drugs is a significant risk for the global effort to reduce disease burden facing the world. Currently, dihydroartemisinin-piperaquine (DHA-PPQ) is the most common drug used to treat *P. falciparum*, but little evidence about the resistance status targeting DHA (ACT drug) and its partner drug (PPQ) has been reported in Shandong Province, China.

**Methods:**

A retrospective study was conducted to explore the prevalence and spatial distribution of *Pfk13* and *Pfcrt* polymorphisms (sites of 72–76, and 93–356) among imported *P. falciparum* isolates between years 2015–2019 in Shandong Province in eastern China. Individual epidemiological information was collected from a web-based reporting system were reviewed and analysed.

**Results:**

A total of 425 *P. falciparum* blood samples in 2015–2019 were included and 7.3% (31/425) carried *Pfk13* mutations. Out of the isolates that carried *Pfk13* mutations, 54.8% (17/31) were nonsynonymous polymorphisms. The mutant alleles A578S, Q613H, C469C, and S549S in *Pfk13* were the more frequently detected allele, the mutation rate was the same as 9.7% (3/31). Another allele *Pfk13* C580Y, closely associated with artemisinin (ART) resistance, was found as 3.2% (2/31), which was found in Cambodia. A total of 14 mutant isolates were identified in Western Africa countries (45.2%, 14/31). For the *Pfcrt* gene, the mutation rate was 18.1% (77/425). T_76_T_356_ and T_76_ were more frequent in all 13 different haplotypes with 26.0% (20/77) and 23.4% (18/77). The CVIET and CVIKT mutant at loci 72–76 have exhibited a prevalence of 19.5% (15/77) and 3.9% (3/77), respectively. The CVIET was mainly observed in samples from Congo (26.7%, 4/15) and Mozambique (26.7%, 4/15). No mutations were found at loci 97, 101 and 145. For polymorphisms at locus 356, a total of 24 isolates were identified and mainly from Congo (29.2%, 7/24).

**Conclusion:**

These findings indicate a low prevalence of *Pfk13* in the African isolates. However, the emergence and increase in the new alleles *Pfcrt* I356T, reveals a potential risk of drug pressure in PPQ among migrant workers returned from Africa. Therefore, continuous molecular surveillance of *Pfcrt* mutations and in vitro susceptibility tests related to PPQ are necessary.

## Background

Imported *Plasmodium falciparum* malaria from Africa, has become a great threat to malaria elimination in China [1]. In 2015–2019, 9702 cases of imported *P. falciparum* malaria have been reported in China, the top five countries of origin are Nigeria, Angola, Ghana, Cameroon and Equatorial Guinea, accounting for 60.4% (5863/9702) [2]. Artemisinin-based combinations are the first-line drugs used to treat uncomplicated *P. falciparum* as recommended by the World Health Organization (WHO) [3]. However, the emergence and spread of *P. falciparum* resistant to artemisinin and partner drugs used in ACT is a significant risk for the global effort to reduce disease burden facing the world, especially for the Greater Mekong Subregion (GMS) and Africa [4, 5]. Molecular marker studies identify and track the prevalence of key molecular mutations [6]. For example, the *P. falciparum kelch-13* (*Pfk13*) gene, served as a molecular marker for artemisinin resistance (ART-R) isolates in a laboratory-based in vitro evolution study which was first observed at the Thai–Cambodia border in 2014, has spread to five countries in the GMS [4, 7]. So far, a total of 10 of the *Pfk13* single nucleotide polymorphisms (SNPs) have been validated in vitro and in vivo as associated with delayed clearance following ACT [8]. Surveillance of *Pfk13* polymorphisms associated with ART-R has also been undertaken in Africa. Recent publications indicated that the clonal expansions of R561H, which was associated with delayed parasite clearance among patients treated with ACT, have been detected in Rwanda [9]. In Uganda, C469Y and A675V, the candidate markers of ART-R, were also found in more than 15% of samples from 2018 to 2019 [10]. Similarity, the R561H was also identified as the main mutation site in Zhejiang Province among migrant workers from Rwanda [11]. In 2017, a patient infected with *P. falciparum* who had returned from Equatorial Guinea reported in Jiangsu Province, was found to harbour the *Pfk13* M579I site, which was confirmed to be linked to ART-R with a 2.29% in vitro survival rate by ring-stage survival assay [12]. In addition, *P. falciparum* resistant to chloroquine (CQ), amodiaquine (AQ), or piperaquine (PPQ) harbour mutations in the *P. falciparum* chloroquine resistance transporter (*Pfcrt*), a transporter resident on the digestive vacuole membrane that can transport those weak-base 4-aminoquinoline drugs out of this acidic organelle. The *Pfcrt* gene K76T mutation, has been confirmed to be closely associated with CQ resistance [13, 14]. However, several novel mutations in *Pfcrt* were found to be associated with PPQ reduced susceptibility [15–17].

In Cambodia, Agrawal et al. [18] identified the locus F145I associated with a decrease in PPQ susceptibility. In a context of dihydroartemisinin-piperaquine (DHA-PPQ) resistance in Cambodia, novel *Pfcrt* mutations such as H97Y, M343L, and G353V were revealed to induce in vitro PPQ resistance [15]. Also treatment failures with DHA-PPQ were associated with T93S, H97Y, F145I and I218F mutations in *Pfcrt* and with plasmepsin 2/3 amplification in Cambodia, Thailand and Vietnam [16, 17]. Besides, the mutation I356T/L is often found both on Asian or South-American parasites [18–20].

Malaria was once endemic in the whole province, though no indigenous case reported in Shandong Province since 2012, whereas the imported *P. falciparum* has increased gradually. For example, the number of imported *P. falciparum* were 857 cases reported in Shandong in 2015–2019, accounting for nearly 8.8% of the total imported *P. falciparum* cases (n = 9702) nationwide.

The national drug policy of China was updated in 2006, since then ACT has been used as the first-line treatment for uncomplicated falciparum malaria, including DHA-PPQ, artesunate-amodiaquine (AS-AQ), artemisinin-naphthoquine phosphate (ART-NQ), and artemisinin-piperaquine (ART-PPQ). Currently, DHA-PPQ is the most common drugs used to treat *P. falciparum* and the resistance status targeting DHA and its partner drug (PPQ) needs to be understood. Therefore, in this study, *Pfk13* and *Pfcrt* mutations in imported Africa and Southeast Asia patients reported in Shandong Province in eastern China from 2015 to 2019 were characterized. This study aimed to investigate and analyse the prevalence of these two gene biomarkers, of which mutant alleles confer to DHA-PPQ resistance.

## Methods

### Study sites and samples

The *P. falciparum* positive samples in this study were collected from symptomatic patients prior to anti-malarial treatment in Shandong Province from 2015 to 2019. The imported cases refer to the malaria cases or infections in which the infection was acquired outside the area in which it was diagnosed. In this study, it refers to the patient who acquired the illness from a known malaria-prevalent region outside China [21]. Individual epidemiological information was also collected from a web-based reporting system (China Information System for Diseases Control and Prevention) and analysed in this study.

A total of 425 *P. falciparum* blood samples from the migrant people who returned from Africa and Southeast Asia to Shandong in 2015–2019 were obtained and examined at enrollment. All the samples were microscopic and PCR positive for *P. falciparum* and were obtained from each patient. Demographic data of all cases including age, sex, occupation, the date of onset, interval from onset to confirmed diagnosis, interval from confirmed diagnosis to report, and source countries were recorded. For each specimen, approximately 200 µl of blood was collected from a finger prick and spotted on a piece of 3MM Whatman filter paper (GE Healthcare, Boston, MA, USA), which was allowed to air-dry. Each of the samples was labeled with a study number and stored at − 20 °C until extraction. The *P. falciparum* genomic DNA from approximately 20 µl of each dried blood sample was then extracted with a QIAamp DNA blood kit (QIAGEN, Valencia, CA) according to the manufacturer’s instructions.

### Treatment and follow-up

Patients were treated by dihydroartemisinin and piperaquine according to the national anti-malarials regulation with a total adult dose of 2.5 mg/kg DHA and 20 mg/kg PPQ for 3 days. For follow-up, the thick and thin blood smear of the patients stayed in this hospital for treatment on Day 3 was collected. Giemsa-stained blood slides were prepared for *Plasmodium* speciation. Slides were examined and read by an expert microscopist certified as Level 1 by the WHO.

### Molecular marker polymorphisms

To investigate polymorphisms in the *Pfk13* (PF3D7_1343700), the *Pfk13* gene were determined by nested PCR amplification of an 849-bp fragment (from amino acids 427–709) as described previously [4]. As for the *Pfcrt* (PF3D7_0709000), the primers for 72–76 sites of the *Pfcrt* gene were subjected to nested PCR amplification [22]. For the 93–356 sites of the *Pfcrt* gene, after 1 round of PCR amplification, the expected PCR product were obtained by 1.5% agarose gel electrophoresis as reported previously [23] and sent for Sanger sequencing (Shanghai BioTechnologies Co., Ltd., Shanghai, China).

### Data analysis

Multiple nucleotide sequence alignments and analysis were performed using the DNAMAN software editor (https://www.lynnon.com/pc/framepc.html). Sequences were analyzed with the Blast online program (http://blast.ncbi.nlm.nih.gov/) using *P. falciparum* 3D7 strain as the reference control. R (Version 4.0.2) statistical software (R Foundation for Statistical Computing, Vienna, Austria) was adopted to conduct statistical analyses.

### Ethical considerations

This study was reviewed and approved by the ethical committee of the National Institute of Parasitic Diseases, Chinese Centre for Disease Control and Prevention (NIPD, China CDC, No. 2019008).

## Results

### Demographics of *P. falciparum* infection

**Fig. 1:**
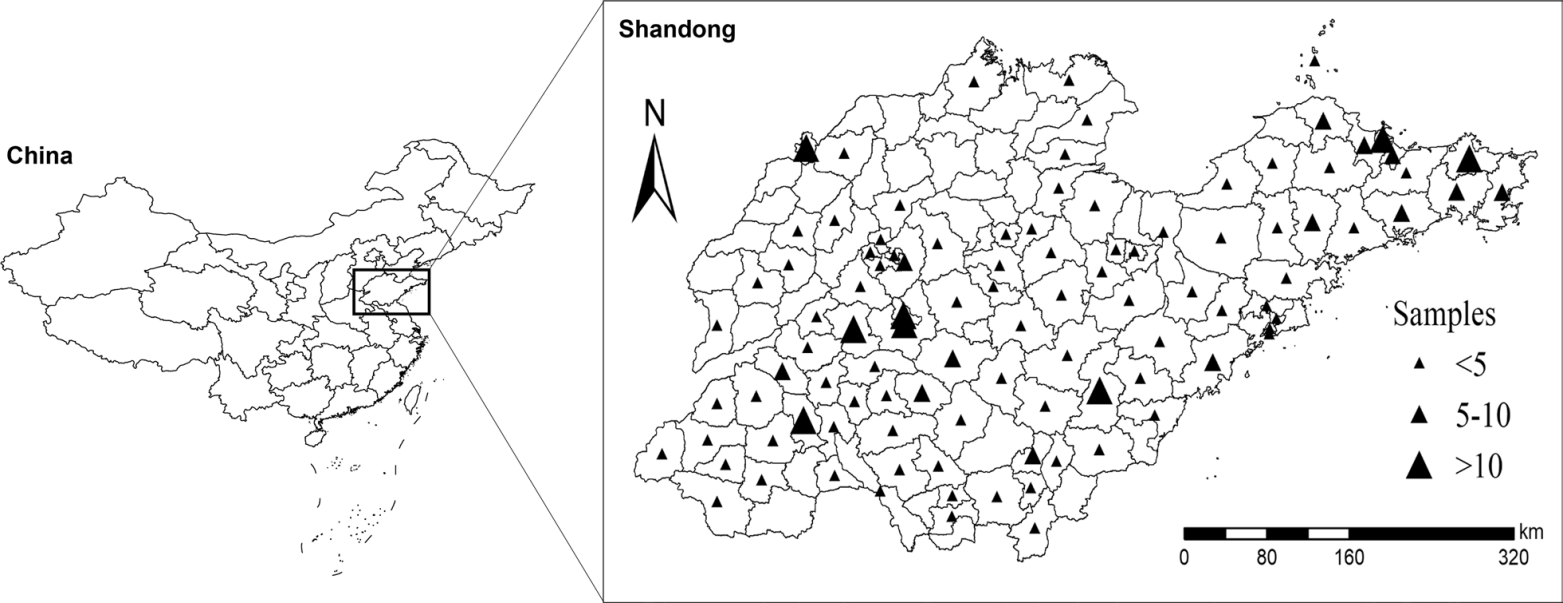
Study sample sites in Shandong. All counties are labelled according to the number of obtained samples using ArcGIS 10.1

A total of 425 *P. falciparum* blood samples were involved in this study. Among all the participants, 418 (98.4%) were male. The patients were mainly found in the age group of 40–50 years old (n = 182, 42.8%) and the median age was 42 years old. The samples were mainly collected in Huancui (n = 21, 4.9%), Feicheng (n = 20, 4.7%), and Zhifu (n = 20, 4.7%; Fig. 1). The most common occupation was farmers (n = 196, 46.1%; Table 1). The imported *P. falciparum* cases were mainly reported in January (n = 52, 12.2%) and October (n = 50, 11.8%). The median (range) cases reported per month was 35 (20–52). The median interval from onset to confirmed diagnosis was 3.4 days, and the median interval from confirmed diagnosis to report was 1 days, respectively. A total of five deaths attributing to the *P. falciparum* were reported in the timeframe but no mutation alleles were detected in *Pfk13* and *Pfcrt*. The *P. falciparum* cases were mainly imported from Nigeria (n = 88, 20.7%), Equatorial Guinea (n = 63, 14.8%), and the Democratic Republic of the Congo (Congo DRC) (n = 61, 14.4%).

**Table 1:** Characteristics of study participants with *P. falciparum* by study sites in Shandong Province, 2015–2019

General	Participants	Proportion (%)
Sex		
Male	418	98.4
Female	7	1.6
Age		
<20	2	0.5
20–30	73	17.2
30–40	102	24.0
40–50	183	43.1
50–60	59	13.9
60–70	5	1.2
>70	1	0.2
Occupation		
Farmer	196	46.1
Worker	117	27.5
House servant	30	7.1
Business	16	3.8
Students	3	0.7
Others	63	14.8

### Molecular analysis of *Pfk13* polymorphisms

**Fig. 2:**
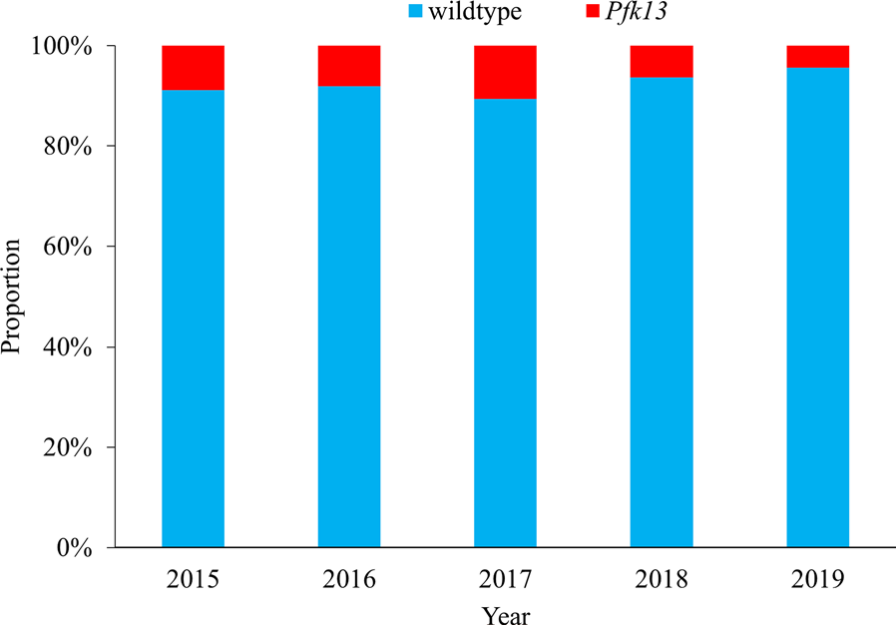
The proportion of isolates of wildtype and *Pfk13* mutations reported in Shandong, 2015–2019

**Fig. 3:**
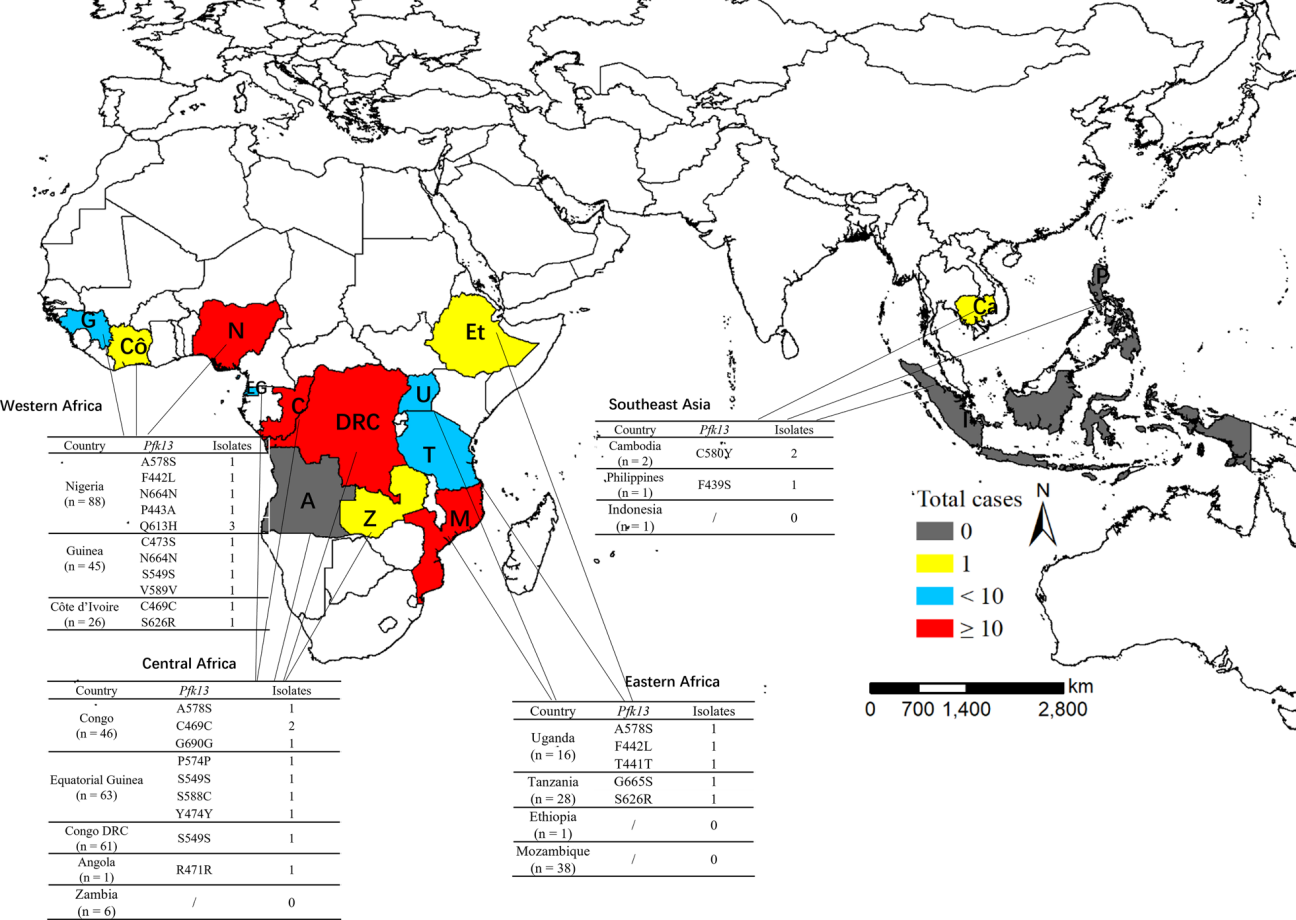
Geographical distribution of *Pfk13* alleles in imported *P. falciparum* isolates. The isolates returned from Eastern Africa, Western Africa, Central Africa, and Southeast Asia are shown in different colours, respectively. The *Pfk13* alleles and number of isolates in each country were listed

Out of all successful sequenced 425 *P. falciparum* isolates, a total of 31 (7.3%) isolates harboring the *Pfk13* polymorphisms were identified in the returners. Among them, 17 (54.8%) were nonsynonymous polymorphisms, whereas the other 14 (45.2%) were synonymous polymorphisms, the ratio of nonsynonymous isolates to synonymous isolates was 1.2. The mutant allele A578S, Q613H, C469C, and S549S were the more frequently detected alleles, and the prevalence was all the same as 9.7% (3/31). The prevalence of mutant allele A578S and Q613H was the same as 17.6% (3/17) in nonsynonymous polymorphisms (Table 2). The *Pfk13* mutation rate was found as 8.9% (4/45), 8.1% (5/62), 10.6% (10/94), 6.3% (7/111), and 4.4% (5/113) in 2015–2019, respectively (Fig. 2). Refer to the geographical distribution of *Pfk13* alleles, Western Africa was the most reported region (n = 14) with Nigeria (n = 8), Guinea (n = 4) and Côte d’Ivoire (n = 2; Fig. 2). In addition, 2 isolates of C580Y were identified among the returners from Cambodia back to Shandong (Fig. 3).

**Table 2:** Nonsynonymous and synonymous SNPs in the *Pfk13* in Shandong Province, 2015–2019

Type of polymorphisms	Genotype	Subtotal	Amino acid reference	Nucleotide reference	Amino acid mutation	Nucleotide mutation^*a*^	Prevalence (%)
Nonsynonymous (n = 17)^b^	F_439_S	1	F	TTT	S	T**C**T	0.2
	F_442_ L	2	F	TTC	L	TT**A**	0.5
	P_443_ A	1	P	CCA	A	**G**CA	0.2
	C_473_ S	1	C	TGT	S	T**C**T	0.2
	A_578_ S	3	A	GCT	S	**T**CT	0.7
	C_580_Y^c^	2	C	TGT	Y	T**A**T	0.5
	S_588_ C	1	S	AGT	C	**T**GT	0.2
	Q_613_ H	3	Q	CAA	H	CA**T**	0.7
	S_626_ R	2	S	AGC	R	AG**A**	0.5
	G_665_ S	1	G	GGC	G	GG**A**	0.2

^a^Mutations are in boldface

^b^The total samples for tested was 425, while 31 of them harboring the *Pfk13* polymorphisms, and 17 were nonsynonymous polymorphisms

^c^Those mutations have been validated as molecular markers associated with in vitro ART-R

### Mutation prevalence of *Pfcrt* polymorphisms

**Fig. 4:**
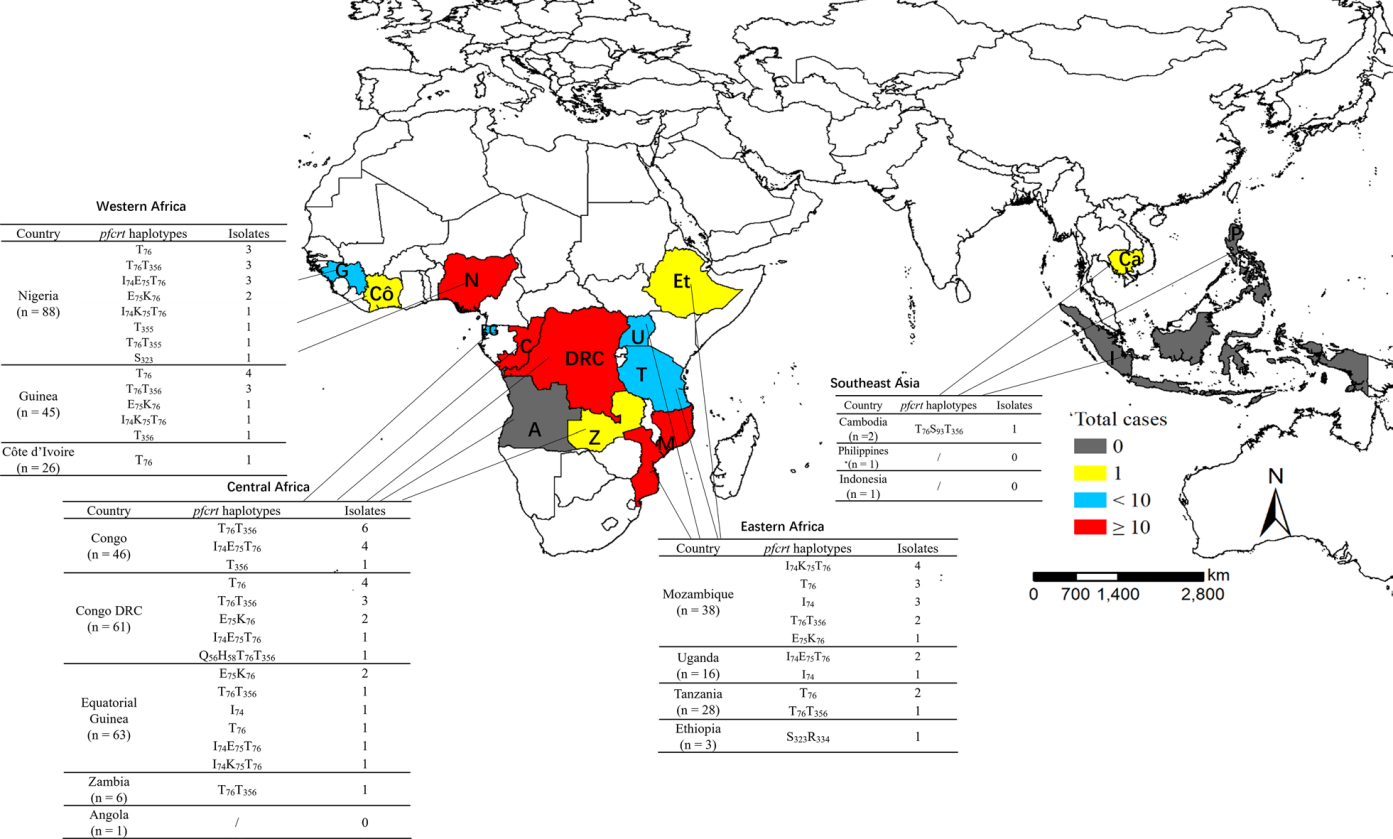
Geographical distribution of *Pfcrt* haplotypes in imported *P. falciparum* isolates. The isolates returned from Eastern Africa, Western Africa, Central Africa and Southeast Asia are shown in different colours, respectively. The *Pfcrt* haplotypes and number of isolates in each country were listed

A total of 13 different haplotypes were identified in 77 isolates (18.1%), including T_76_T_356_ found in 20 isolates (4.7%), and T_76_ in 18 isolates (4.2%) (Table 3). Refer to the codon 72–76, the mutation alleles with CVIET and CVIKT were identified, exhibiting a prevalence of 3.5% and 0.7%, respectively (Table 3). The CVIET were mainly distributed in Congo (5.2%, 4/77) and Mozambique (5.2%, 4/77). In addition, one isolate carrying S_93_ was identified in Cambodia in 2015, and no mutations was found at loci 97, 101 and 145. For the mutation alleles at 323–355, we have identified one isolate harbouring S_323_R_334_ in Ethiopia, and 2 isolates carrying T_355_ and T_76_T_355_, all were from Nigeria. For polymorphisms at locus 356, a total of 24 isolates were identified and most of them were from Congo ( 29.2%, 7/24). The geographical distribution of *Pfcrt* haplotypes showed that 30 isolates and 26 isolates harbouring *Pfcrt* haplotypes were found in Central Africa (39.0%, 30/77) and Western Africa (33.8%, 26/77) (Fig. 4). Interestingly, two *P. falciparum* isolates with *k13* + *crt* polymorphisms were found in the patients returned from Cambodia (C580Y in *Pfk13* and K76TT93SI356T *in Pfcrt*) and Congo (G690G in *Pfk13* and K76I356T in *Pfcrt*).

**Table 3:** Genetic haplotypes of *Pfcrt* with *P. falciparum* in Shandong Province, 2015–2019

Haplotypes^a^	Subtotal^b^	Proportion^c^ (%)
**T**_76_ **T**_356_	20	4.7
**T**_76_	18	4.3
**I**_74_ **E**_75_ **T**_76_	15	3.6
**E**_75_ **K**_76_	8	1.9
**I**_74_	5	1.2
**I**_74_ **K**_75_ **T**_76_	3	0.7
**T**_356_	2	0.5
**T**_355_	1	0.2
**Q**_56_ **H**_58_ **T**_76_ **T**_356_	1	0.2
**T**_76_ **T**_355_	1	0.2
**T**_76_ **S**_93_ **T**_356_	1	0.2
**S**_323_	1	0.2
**S**_323_ **R**_334_	1	0.2

^a^Mutations are in boldface

^b^The total samples for tested was 425, while 77 of them harboring the *Pfcrt* polymorphisms

^c^The proportion refers to the mutated genotype samples accounting for the whole samples collected at the study site

## Discussion

The artemisinin-resistant *P. falciparum* became a great challenge to the countries facing malaria control and elimination. China has eliminated indigenous malaria in 2021 [24], but the imported malaria, especially from Africa and Southeast Asia, has increased significantly [25–27]. Therefore, knowledge of the molecular markers associated with ART-R is crucial for its elimination and post-elimination surveillance. The previous work indicated that low prevalence of *Pfk13* mutations found in the migrant workers from Africa, and A578S was the most common mutation site but showed no relationship with clinical or in vitro ART-R [22, 28, 29]. In this study, the migrant workers with *P. falciparum* infection abroad back to Shandong Province were genetically characterized and the genetic signature at the *Pfk13* and *Pfcrt* candidate drug-resistance marker loci for DHA-PPQ was investigated.

The prevalence refers to SNPs in *Pfk13* was 7.3% in this study, which was slightly higher than that reported in the previous work [22, 28–31]. Except for the A578S, which was considered as the most common *Pfk13* mutant alleles [32, 33], other three alleles including Q613H, C469C, and S549S were still found. The all 3 Q613H were detected in the migrant workers from Nigeria, which was similar as reported before [34]. The synonymous mutant allele C469C, which was common in Africa, was identified in this study and the proportion was similar as reported in Burkina Faso and Senegal [35–37]. The *Pfk13* mutant allele S549S, to our knowledge, was firstly identified in the migrant workers from Guinea, Equatorial Guinea, and Congo DRC. This was not surprising as C580Y was firstly found in the Thai–Cambodia border and now had spread into several counties including Myanmar, Laos, Vietnam and Guyana [38–42]. Therefore, further studies are needed to determine the extent of the spread of the *Pfk13* polymorphisms in Africa, and to investigate any relationships between these mutations and changes to parasite clearance time and in vitro ART-R.

Despite of the high prevalence of *Pfcrt* K76T observed in GMS [43], as well as the China-Myanmar border [44, 45], the low prevalence rate (4.2%) of *Pfcrt* K76T mutation in African countries reported by the present study reflects high susceptibility of *P. falciparum* to the CQ. The codons at 72–76 with CVIET was the dominant mutant haplotypes in *Pfcrt* genotype and it was commonly found in African isolates [46–48]. Similarity, CVIET was mainly distributed in Central Africa (7.8%) and Eastern Africa (7.8%). SVMNT was mainly identified in Southeast Asia but not found in the current study. In addition, DHA + PPQ was once highly effective and adopted as the first-line anti-malarial in Cambodia, Vietnam, and Thailand, but due to the multidrug-resistant PfPailin lineage came to dominate in the GMS [49], high rates of treatment failure occurred. Studies indicated that treatment failure increased with the acquisition of mutations in *Pfcrt* mutation of S93, 97Y, 101 F, 145I, 343 L, 353 V, and 356T, which reduced piperaquine susceptibility [15, 50–52]. The 356T mutation was found with a high prevalence of 36.5% in Congo in the year of 2011–2012 [53]. However, the lower mutation result of T_76_T_356_ mixed type was 4.7%, which was reported in a few studies [23, 50]. Therefore, continuous molecular surveillance of *Pfcrt* mutations and in vitro susceptibility tests related to PPQ are necessary, especially for the migrant workers returning from African countries.

There are two limitations in this study. Firstly, no clinical data were included in this study, which may not provide the entire information for the molecular epidemiological analysis. Secondly, the molecular investigations on *Pfk13* and *Pfcrt* were only conducted, but lack data on *Pfmdr*1 and *Pfplasmepsin* 2 and 3 genes due to the financial limitations. In the next phase of this study, those two genes SNP will be looked at.

## Conclusion

This study identified the prevalence and spatial distribution of molecular markers *Pfk13* and *Pfcrt* from imported *P. falciparum* isolates in Shandong Province. The findings suggest that a low mutation rate of *Pfk13* was observed and mainly clustered in the Western and Central Africa. In addition, the low prevalence *Pfcrt* K76T point mutation in African countries reflects high susceptibility of *P. falciparum* to the CQ. However, the increase in the new alleles *Pfcrt* I356T, reveals a potential risk of drug pressure in PPQ. Therefore, it is imperative to carry out continuous surveillance of molecular markers among those migrant workers and explore in vitro relationship of ART-R with the clinical trials.

## Data Availability

The datasets analysed in this study are available from the corresponding author on reasonable request.
